# Does Health Professional Counseling Impact the Quality-of-Life Levels of Older Adults Enrolled in Physical Activity Programs?

**DOI:** 10.3390/medicina56040146

**Published:** 2020-03-25

**Authors:** Claudio de Lira, Henrique Taveira, Weverton Rufo-Tavares, Douglas Santos, Paulo Celini, Lucas Oliveira, Marilia Andrade, Pantelis Nikolaidis, Thomas Rosemann, Beat Knechtle, Rodrigo Vancini

**Affiliations:** 1 Faculdade de Educação Física e Dança, Universidade Federal de Goiás, Goiânia 74690-900, Brazil; 2 Centro de Educação Física e Desportos, Universidade Federal do Espírito Santo, Vitória 29075-910, Brazil; henrique.gigante191@hotmail.com (H.T.); wevertonrts@hotmail.com (W.R.-T.); paulo.celini@hotmail.com (P.C.); lukaas.eckoo@hotmail.com (L.O.); rodrigoluizvancini@gmail.com (R.V.); 3 Colegiado de Educação Física, Universidade do Estado da Bahia, Teixeira de Freitas 45992-255, Brazil; datsantos@uneb.br; 4 Departamento de Fisiologia, Universidade Federal de São Paulo, São Paulo 04023-900, Brazil; marilia1707@gmail.com; 5 Exercise Physiology Laboratory, 18450 Nikaia, Greece; pademil@hotmail.com; 6 Institute of Primary Care, University of Zurich, 8091 Zurich, Switzerland; thomas.rosemann@usz.ch; 7 Medbase St. Gallen Am Vadianplatz, 9001 St. Gallen, Switzerland; beat.knechtle@hispeed.ch

**Keywords:** aging, older people, physical activity, quality of life, exercise

## Abstract

*Background and objectives:* There are studies showing that exercise counseled by health professionals can improve physical fitness. However, less is known about the effects of exercise counseling on quality of life. The aim of this study was to investigate health-related quality of life of older adults who received or did not receive physical exercise counseling by sport and exercise professionals or physicians. *Materials and Methods:* This was a cross-sectional study that investigated quality of life of older adults who did or did not receive exercise counseling from health professionals. Older adults who were physically active took part in this study: 45 participants performed exercise advised by sport and exercise professionals (SEPCG), 19 participants performed exercise advised by physicians (PCG), and 26 participants performed exercise without counseling (NCG). Participants answered the SF-36 to estimate quality of life. *Results:* Analysis revealed that responses on all SF-36 subscales were higher in those participants who received counseling by sport and exercise professionals (Functioning capacity, β = −26.283, p < 0.001 and β = −26.482, *p* < 0.001, Role limitations due to physical problems, β = −43.372, *p* < 0.001 and β = −45.177, *p* < 0.001, Pain, β = −17.634, *p* < 0.001 and β = −16.015, *p* < 0.001, General health perceptions, β = −38.008, *p* < 0.001 and β = −32.529, *p* < 0.001, Vitality, β = −18.573, *p* < 0.001 and β = −16.406, *p* = 0.001, Social functioning, β = −37.963, *p* < 0.001 and β = −29.224, *p* < 0.001, Role limitations due to emotional problems, β = −52.246, *p* < 0.001 and β = −40.173, *p* < 0.001, Mental health, β = −17.381, *p* < 0.001 and β = −12.121, *p* < 0.001, PCG and NCG respectively). *Conclusions:* The results showed that those older adults who were counseled by sport and exercise professionals presented better quality of life, possibly because these professionals counseled exercise based on current guidelines for exercise prescription.

## 1. Introduction

Health-related quality of life is associated with individual perception about conditions that can affect health status and other aspect of live, including work, leisure activities, and social relationships [1,2]. In older adults, noncommunicable diseases associated with aging (for example: diabetes mellitus, arterial hypertension and mental disorders) may be detrimental for quality of life. Therefore, strategies that can improve the quality of life should be adopted. Corroborating this view, many older adults with noncommunicable diseases prefer quality of life to longevity [3]. 

In this context, physical activity can be an effective means of prevention and therapy of many noncommunicable diseases that impact the quality of life of older people [4,5,6]. Indeed, increased physical activity in older adults has been demonstrated to be predictive of better future physical function and self-efficacy [7]. In addition, Rejeski et al. [8] have demonstrated that physical activity improves the overall quality of life despite age, activity status, or the health of older adults.

Previously, cross-sectional data have shown a positive effect of physical activity on health-related quality of life [9]. In elderly, there is a positive association between physical activity and quality of life that varies according to the domain analyzed [4]. In the study conducted by Puciato et al. [5], higher average rates of general quality of life, perceived health status, and quality of life in the physical, psychological, social and environmental domains were demonstrated by the interviewees whose intensity of physical activity was higher. Despite these inspiring findings, around 31% of adults were insufficiently active [10]. Specifically, with regards to older adults, sedentary behavior is greater than in younger people [11,12]. Altogether, these data urge increased participation of older adults in regular physical activity programs.

Although the benefits to older adults of chronic physical activity are incontestable, it is rational to assume that the benefits of physical activity are maximized when properly counseled. Therefore, counseling about exercise prescription provided by specialized personnel, such as sport and exercise professionals (also called physical education professionals or kinesiologists in some countries), is desirable. Indeed, these professionals have a solid academic background in physical training, physical fitness assessment, and exercise prescription. Furthermore, several factors are associated with the practice of physical activity, among which motivational factors stand out [13,14,15,16,17]. Therefore, regular monitoring of physical activity practice by a professional seems to be a great source of motivation [18,19,20]. Again, the sport and exercise professionals have knowledge to advise people on how to exercise safely [21].

Conversely, other health professional, such as physicians and nurses may be an important trigger to change the population`s lifestyle, including physical activity levels. Indeed, as most people visit physicians each year [22] and have confidence in the information provided by them, physicians have the potential to cause change in the patient lifestyle. Despite other health professionals may be important agents to trigger changing in physical exercise levels, a study found that only 19% of physicians provide counseling for patients about exercise [23]. Conversely, Jones et al. [24] found that only 30-second verbal exercise recommendation made by physician significantly increase the amount of exercise among cancer patients. However, other studies did not find effectiveness of counseling for exercise made by physicians for physical activity variables [25,26,27,28] and health status [25,26,27,28]. Altogether, these studies suggest that results concerning the effectiveness of physician counseling for exercise are ambiguous, probably because physicians do not have academic background about exercise science and because physicians are not interested in exercise science [29]. Reinforcing the idea that exercise should be prescribed and supervised by sport and exercise professionals.

Given that health-related quality of life is an important construct for older adults and that the demonstration of a positive association between physical activity level and health-related quality of life, the aim of this study was to investigate health-related quality of life of older adults who received or did not receive physical exercise counseling by sport and exercise professionals or physicians in real-world settings. Because it has already been shown that social characteristics (schooling and economic class) can influence quality of life [30,31], we also evaluated these characteristics in the sample investigated. We hypothesized that those who received physical exercise counseling by sport and exercise professionals will present higher health-related quality of life than those who received physical exercise counseling by physicians and then those who did not receive counseling, due to the solid academic background that sport and exercise professionals possess about exercise science. In practical terms, this study highlights the importance of counseling by sport and exercise professionals to increase health-related quality of life of the older adults and results from current study can be used by primary healthcare settings to improve exercise counseling directed to older people.

## 2. Materials and Methods

### 2.1. Participants

This cross-sectional study involved 90 older adults from both sexes (a convenience sample, 47 males), aged between 60 and 75 years, who were apparently healthy, who were recruited using advertisements placed in private and public exercise facilities. Achieved statistical power was calculated post-hoc using the G-Power algorithm: F tests—Linear multiple regression: Fixed model, R^2^ increase. The effect size was computed from measured total score of quality of life as R^2^ = 0.318. For effect size = 0.46, with α error probability  = 0.05, total sample size = 90, number of tested predictors = 1 and total number of predictors = 4; computed power (1−β error probability)  = 0.99.

The inclusion criteria used was having a body mass index (BMI) lower than 35 kg/m^2^, because previous studies showed that obesity can impair quality of life [32,33]. Sedentary older adults were excluded from the sample. Participants who performed exercise advised by sport and exercise professionals constituted the sport and exercise professional counseling group (SEPCG, *n* = 45, 26 males), those who performed exercise advised by physicians constituted the physician counseling group (PCG, *n* = 19, 10 males), and participants who performed exercise without counseling from either sport and exercise professionals or physicians constituted the non-counseling group (NCG, *n* = 26, 11 males). The information about whether participants were counseled or not was collected by self-report. After an explanation of the purposes of study, written consent was obtained. The age (years) of the participants of SEPCG, PCG, and NCG was 65.0 (61.5–67.0) (medians (Quartile 1–Quartile 3)), 64.0 (60.0–65.0) and 65.0 (60.0–67.3), respectively. The height (cm) of the participants of SEPCG, PCG, and NCG was 169.0 (161.5–173.0), 163.0 (155.0–169.0) and 165.5 (159.0–170.0), respectively. The body mass (kg) of the participants of SEPCG, PCG, and NCG was 72.0 (59.0–79.0), 75.0 (60.0–82.0) and 73.5 (67.5.0–82.0), respectively. The BMI (kg.m^−2^) of the participants of SEPCG, PCG, and NCG was 24.8 (21.5–26.1), 27.5 (22.8–28.7) and 27.5 (24.1–29.9), respectively. Ethics approval of research was granted by the University Human Research Ethics Committee (#2.186.599 with date of approval 26 July 2017).

### 2.2. Counseling and Non-Counseling Groups

The SEPCG received exercise counseling by sport and exercise professionals. Briefly, the exercise counseling involved orientation about exercise intensity, duration of session, and weekly frequency. Sport and exercise professionals prescribed exercise based on the current guidelines of the American College of Sports Medicine (ACSM) [34]. Therefore, it is reasonable to suppose that sport and exercise professionals ensured the right dose and progression of exercise training.

The PCG received exercise counseling by physicians. Physicians advised participants only to perform brisk walking without counseling about exercise intensity, frequency, and duration. The NCG did not receive exercise prescription and/or supervision. Participants from the NCG performed only walking. It is important to emphasize that the present study had a “real-world” approach. Therefore, each volunteer had his or her own training routine. 

### 2.3. Variable Measurement and Instrumentation

Participants answered four questionnaires: (a) a questionnaire for collecting demographic data (age, gender, work status, and main occupation, educational achievement, and marital status), (b) a questionnaire for collecting exercise routine data, (c) a questionnaire to assess economic level, and (d) the SF-36 (Medical Outcomes Study 36-Item Short-Form Health Survey) to evaluate their health-related quality of life.

The exercise routine questionnaire comprised four open questions intended to capture information on training experience, whether receiving counseling from a sport and exercise professional or physician, weekly volume and frequency, and which kind of physical activity was engaged.

Economic level was defined according to the Brazilian Economic Classification Criteria of the Brazilian Association of Research Companies (ABEP) [35]. This questionnaire estimates the purchasing power of participants by the sum of the values of owned items plus the sum of the level of education, generating a score. In accordance with this score, the participants were classified in following economic classes (in decreasing order of purchasing power): stratum A1 (score: 42–46 points), stratum A2 (score: 35–41 points), stratum B1 (score: 29–34 points), stratum B2 (score: 23–28 points), stratum C1 (score: 18–22 points), stratum C2 (score: 14–17 points), stratum D (score: 8–13 points), and stratum E (score: 0–7 points). Participants from classes A and B were classified as high economic level, those from class C as intermediate economic level, and those from classes D and E as low economic level. The internal consistency of the ABEP questionnaire is good, with Cronbach’s alpha ranging from 0.78 to 0.82 [36].

The Medical Outcomes Study 36-Item Short-Form Health Survey (SF-36), translated and validated for Brazilian Portuguese, was used to assess health-related quality of life [37]. The questionnaire has 36 items in eight subscales. The subscales are: functioning capacity (ten items), role limitations due to physical problems (four items), pain (two items), general health perceptions (five items), vitality (four items), social functioning (two items), role limitations due to emotional problems (three items), and mental health (five items). Furthermore, a single item provides an indication of change in health for 12 months. Subscale scores range from 0 to 100, the higher the score, the better is the quality of life. The questionnaire was conducted as an interview format to avoid misinterpretation. Cronbach’s alpha of SF-36 ranging from 0.76 to 0.90 for all subscales, therefore the internal consistency of the SF-36 is good [38].

### 2.4. Statistical Analyses 

The Shapiro–Wilk test revealed that the continuous variables (age, height, body mass, BMI, and characteristics of the physical training) were not normally distributed. For this reason, the data were expressed as medians (Quartile 1–Quartile 3), and the Kruskal-Wallis test followed by Dunn’s multiple comparison procedure was used to compare groups (SEPCG, PCG, and NCG). Categorical variables (work status, educational status, marital status, and economic class) were described as frequencies and percentages. Each subscale of SF-36 of the SEPCG were compared to the PCG and NCG, using the multiple linear regression model (enter method) for adjustment, considering the variables (age, sex, economic class, and educational status) [30,31]. SPSS software (version 20.0, IBM Corp., USA) was used to run statistical analyses. The level of significance was considered to be 5%.

## 3. Results

The Kruskal-Wallis test followed by Dunn’s multiple comparison procedure was used to verify differences in age, height, body mass, and BMI. The participants from SEPCG presented lower BMI as compared with participants from PCG and NCG (*p* = 0.001). 

Descriptive sociodemographic data are presented in Table 1. We found that 87.8% of participants did not work, 38.9% had not completed elementary school, 83.3% were married, and 62.2% were from the intermediate economic class. 

The characteristics of physical training are presented in Table 2. The Kruskal-Wallis test followed by Dunn’s multiple comparison procedure demonstrated a significant difference between SEPCG and PCG in daily volume of exercise (*p* = 0.009) and a significant difference in SEPCG vs. PCG and NCG in weekly frequency (*p* < 0.0001) and weekly volume of exercise (*p* < 0.0001). There was no difference among groups in exercise experience (*p* = 0.67).

In addition, of the 45 participants from SEPCG, 57.8% (*n* = 26) practiced walking and resistance training, 4.4% (*n* = 2) practiced walking and Pilates, 6.7% (*n* = 3) practiced walking and water aerobics, 6.7% (*n* = 3) practiced resistance training and cycling, 15.6% (*n* = 7) practiced resistance training, and 8.9% (*n* = 4) practiced Pilates.

Table 3 presents the multivariate linear regression analyses to predict SF-36 subscales based on adjusted for all potential confounders. Significant regression equations were found for the functioning capacity((F(6,83) = 8.924; *p* < 0.001; R^2^ = 0,348)), role limitations due to physical problems ((F(6,83) = 28.797; *p* < 0.001; R^2^ = 0.652)), pain ((F(6,83) = 7.212; *p* < 0.001; R^2^ = 0.295)), general health perceptions((F(6,83) = 12.657; *p* < 0.001; R^2^ = 0.440)), vitality ((F(6,83) = 5.039; *p* < 0.001; R^2^ = 0.214)), social functioning((F(6,83) = 13.287; *p* < 0.001; R^2^ = 0.453)), role limitations due to emotional problems((F(6,83) = 24.753; *p* < 0.001; R^2^ = 0.616)) and mental health (F(6,83) = 5.734; *p* < 0.001; R^2^ = 0.242).

In practical terms, participants from PCG and NCG were significantly associated with lower quality of life for all SF-36 subscales. Specifically, PCG and NCG presented functioning capacity subscale score about 26 points lower than SEPCG. PCG and NCG presented role limitations due to physical problems scores about 43 and 45 points lower than SEPCG, respectively. PCG and NCG presented pain scores about 18 and 16 points lower than SEPCG, respectively. PCG and NCG presented vitality scores about 19 and 16 points lower than SEPCG, respectively. PCG and NCG presented social functioning scores about 38 and 29 points lower than SEPCG, respectively. PCG and NCG presented role limitations due to emotional problems scores about 52 and 40 points lower than SEPCG, respectively. PCG and NCG presented mental health scores about 17 and 12 points lower than SEPCG, respectively.

## 4. Discussion

Previous evidence supports that counseling, advice, and/or supervision from specialized healthcare personnel can increase the positive impact of physical training on physical fitness [39,40]. However, no study has investigated the effect of physical exercise counseling by health professionals on quality of life in older adults. The main aim of this study was to assess whether health-related quality of life of physically active older adults who receive counseling by sport and exercise professionals is significantly associated with higher quality. The main result was that the quality of life of the physically active older adults who were counseled by sport and exercise professionals was higher as compared with those counseled by physicians and those without counseling. Therefore, the results indicate that counseling about physical exercise from sport and exercise professionals contributed to better health-related quality of life.

Previously, Buttery and Martin [41] found that older adults have poor knowledge about the benefits of physical exercise. Therefore, it is reasonable to assume that when these older frail adults are counseled by health professionals, the benefits, knowledge, and attitudes of exercise can be enhanced and clarified. Indeed, feedback and discussion are important strategies to clarify people about particular subject, such as benefits of physical exercise and related themes. Thus, it is reasonable to assume that sports and exercise professionals have more academic background to meet these requirements, as compared with physicians and other health professionals working in primary healthcare.

To show the benefits of supervision, Gentil and Bottaro [40] investigated the effect of different supervision ratios (high, 1:5 professional-to-participants ratio; and low, 1:25 supervision ratio) on muscle strength. The authors showed that the magnitude of muscular strength gains was higher in participants training under higher supervision ratios, due probably to the higher exercise intensity. Therefore, this is a possible explanation of the difference obtained between groups. Albeit speculative, it is rational to suppose that exercise intensity performed by SEPCG was higher than the other two groups because, as mentioned in the methods section, sport and exercise professionals advised and counseled exercise based on the current guidelines of the ACSM. 

Previously, systematic reviews have investigated the effect of exercise intensity on quality of life. For example, Netz et al. [42] found better quality of life as resulted of moderate-intensity exercise as when compared with light or strenuous intensity exercise. Therefore, exercise intensity may be particularly pertinent to quality-of-life outcomes. Therefore, it is noteworthy that sport and exercise professionals had the academic background to follow these recommendations.

Another possible explanation for effectiveness of sport and exercise counseling on quality of life is the quantity of exercise. According to the ACSM [34], physical activity of moderate intensity of 150 min/week is associated with improvement of cardiorespiratory fitness and health. With regard to exercise volume, we found that SEPCG attained 120 min/week (median), which is close to the ACSM´s guidelines, while PCG and NCG exercise volume corresponds to 60 and 90 min/week, respectively (median). Alternatively, the ACSM also recommends vigorous-intensity physical exercise performed ≥3 days/week or for a total of ~75 min/week. However, considering the nature of the physical activities performed by the sample (e.g., walking), it is not probable that these activities can attain vigorous level. Thus, volume of exercise below current recommendations may be also a possible explanation for lower quality of life found in groups not counseled by sport and exercise professionals.

Corroborating this hypothesis, Brown et al. [9] found that recommended levels of physical activity are associated with better overall quality of life and perceived status. In our sample, the PCG and NCG did not follow current recommendations about exercise prescription. Therefore, this could be an explanation, because quality of life of PCG and NCG was lower than SEPCG. 

In another study, Fennell et al. [43] found a significant increases in level of physical activity and muscular endurance when training intervention was supervised by exercise professionals; however, when receiving only recommendations for the practice of physical activity without supervision, the participants returned to baseline scores, losing the acquired effects. Probably these results can be explained by motivational factors [44,45]. In this context, it was demonstrated that high level of satisfaction can be reached in activities that are more pleasant cause highest levels of life satisfaction [46]. Therefore, enjoyment plays a key role in satisfaction with involvement in physical activity. These findings that enjoyment may be a possible mediator in life satisfaction with involvement in physical activity. Indeed, quality of life is influenced by social variables, such as the social context of physical activity [47]. This may be another reasonable factor to explain the higher health-related quality of life found in SEPCG. Indeed, most of the participants from SEPCG performed other activities than a simple walk, such as Pilates and water aerobics, and previous studies had already demonstrated that these kinds of activities are considered attractive and enjoyable [48,49,50].

In regard to type of exercise, previous studies showed that people that performed only walk had similar health-related quality of life compared with the sedentary group [51,52]. Again, participants from SEPCG performed other activities beyond walking, and these also can be an explanation of current results.

One alternative to counsel given by sports and exercise professionals is to train physicians about the health-related benefits of exercise. In this context, Marcus et al. [53] investigated the effects of a two-hour training activity offered for physicians that was directed increase knowledge about physical exercise and to increase ability in counseling techniques. The authors found that training method used increased physical exercise knowledge. Considering that counseling/prescribing exercise require special knowledge about exercise physiology and physical training, these results strengthen the need for education programs directed to physicians to raise the level of knowledge about exercise science. Finally, participants from SEPCG presented lower BMI as compared with the two other groups. Previous studies have shown that obesity is negatively associated with quality of life [54].

### Limitations of Study

The study had some limitations which we will enumerate here. First, it is difficult to establish a causal relationship between quality of life and exercise counseling at present study because of the nature of cross-sectional studies. Thus, it is impossible to determine whether subjects advised by sports and exercise professional present higher quality of life or if higher quality of life is responsible for participants seeking counseling by sports and exercise professionals. Second, exercise intensity was not quantified. Third, some researchers suggest that the benefits of physical exercise on quality of life may depend on the presence of chronic diseases [55,56]. Unfortunately, these data were not collected in the current study. Fourth, compliance levels of participants with exercise program were not assessed. Fifth, the sample in our study was small and some caution is required in interpretation. Sixth, the sport and exercise professionals and physicians were not assessed. Seventh, it is not possible to establish cause-effect relationship, since this was a cross-sectional study. Therefore, longitudinal studies should be conducted. Nevertheless, the present study was conducted in real-world setting and for this reason have high ecological validity.

## 5. Conclusions

The results show that physically active older people who were counseled about physical exercise by sport and exercise professionals presented better health-related quality of life as assessed by SF-36. These results highlight the importance of counseling by specialized healthcare personnel to increase health-related quality of life. Our results may be of importance for public and private health facilities that work with physical activity and the older adults; therefore, we encourage the managers of these places to hire sport and exercise professionals and to ensure that exercise be prescribed by sport and exercise professionals to obtain more exuberant results in the health-related quality of life of the older adults.

## Figures and Tables

**Table 1:** Sociodemographic characteristics and economic class.

	SEPCG (*n* = 45)		PCG (*n* = 19)		NCG (*n* = 26)		TOTAL (*n* = 90)	
	*n*	(%)	*n*	(%)	*n*	(%)	*n*	(%)
**Work**								
Yes	9	20	0	0	2	7.7	11	12.2
No	36	80	19	100	24	92.3	79	87.8
**Education**								
Completed higher education	9	20	0	0	0	0	9	10.0
Incomplete higher education	3	6.7	0	0	0	0	3	3.3
Completed high school	21	46.7	0	0	0	0	21	23.3
Incomplete high school	5	11.1	0	0	0	0	5	5.6
Completed elementary school	5	11.1	5	26.3	7	26.9	17	18.9
Incomplete elementary school	2	4.4	14	73.7	19	73.1	35	38.9
**Marital status**								
Single	0	0	0	0	0	0	0	0
Married	37	82.2	18	94.7	20	76.9	75	83.3
Divorced	3	6.7	0	0	4	15.4	7	7.8
Widower	5	11.1	1	5.3	2	7.7	8	8.9
**Economic class**								
High	32	71.1	1	5.3	1	3.8	34	37.8
Intermediate	13	28.9	18	94.7	25	96.2	56	62.2
Low	0	0	0	0	0	0	0	0

Data are presented as frequencies and percentages. SEPCG: sport and exercise professional counseling group. PCG: physician counseling group. NCG: non-counseling group.

**Table 2:** Characteristics of the physical training reported by participants.

Variables	SEPCG (*n* = 45)	PCG (*n* = 19)	NCG (*n* = 26)	*p* value
Daily volume of exercise (min)	30.0 (30.0–60.0)	30.0 (30.0–30.0) *	30.0 (30.0–37.5)	0.009
Weekly frequency	3.0 (3.0–4.0)	2.0 (2.0–2.0) *	2.0 (2.0–3.0) *	<0.0001
Weekly volume of exercise (min)	120.0 (90.0–180.0)	60.0 (60.0–60.0) *	90.0 (60.0–120.0) *	<0.0001
Exercise experience (months)	17.0 (6.5–48.0)	24.0 (12.0–24.0)	24.0 (12.0–36.0)	0.6703

Data are presented as medians (Quartile 1–Quartile 3). SEPCG: sport and exercise professional counseling group. PCG: physician counseling group. NCG: non-counseling group. * indicates significant differences from SEPCG.

**Table 3:** Multivariate linear regression coefficients for quality-of-life subscales of SF-36.

Variables	SEPCG ***			PCG				NCG				
	β **	*p*	SE	β **	*t*	*p*	CI	SE	β **	*t*	*p*	CI
Functioning capacity	1		6.762	−26.283	−3.887	<0.001	(−39.733 to −12.833)	6.358	−26.482	−4.165	<0.001	(−39.128 to −13.836)
Role limitations due to physical problems			6.272	−43.372	−6.915	<0.001	(−55.848 to −30.897)	5.897	−45.177	−7.661	<0.001	(−56.906 to −33.447)
Pain			3.904	−17.634	−4.517	<0.001	(−25.399 to −9.868)	3.671	−16.015	−4.363	<0.001	(−23.316 to −8.714)
General health perceptions			5.919	−38.008	−6.421	<0.001	(−49.781 to −26.236)	5.565	−32.529	−5.845	<0.001	(−43.598 to −21.460)
Vitality			5.021	−18.573	−3.699	<0.001	(−28.560 to −8.585)	4.721	−16.406	−3.475	0.001	(−25.796 to −7.015)
Social functioning			6.375	−37.963	−5.955	<0.001	(−50.643 to −25.283)	5.994	−29.224	−4.875	<0.001	(−41.146 to −17.301)
Role limitations due to emotional problems			7.190	−52.246	−7.266	<0.001	(−66.547 to −37.944)	6.760	−40.173	−5.942	<0.001	(−53.619 to −26.727)
Mental health			3.954	−17.381	−4.395	<0.001	(−25.246 to −9.516)	3.718	−12.121	−3.260	0.002	(−19.516 to − 4.726)

Adjusted for age, sex, economic class, and educational status. ** β: standardized beta coefficient (β represents the change of the standard deviation in quality-of-life score resulting from a change of one standard deviation in the independent variable). *** Reference category. SEPCG: sport and exercise professional counseling group. PCG: physician counseling group. NCG: non-counseling group.
